# Trends of Mortality in Greece Prior to and During Its Current Financial Crisis (2009–2015)

**DOI:** 10.5041/RMMJ.10368

**Published:** 2019-07-18

**Authors:** Konstantinos Z. Vardakas, Margarita Kyriakidou, Katerina N. Apiranthiti, Spiridoula E. Almpani, Dominiki Heliou, Dimitra Stratigopoulou, Eleni Giourmetaki, Dimitra Lamprou, Georgia Binou, Elena Mpaltzoglou, Matthew E. Falagas

**Affiliations:** 1 Alfa Institute of Biomedical Sciences, Athens, Greece; 2 Department of Internal Medicine, Henry Dunant Hospital Center, Athens, Greece; 3 Department of Applied Mathematical and Physical Sciences, National Technical University of Athens, Athens, Greece; 4 Department of Internal Medicine, Tufts University School of Medicine, Boston, MA, USA

**Keywords:** Birth rate, crisis, Hellas, mortality

## Abstract

**Objectives:**

To study mortality changes in Greece prior to and during the financial crisis.

**Study design:**

Analysis of data by the Hellenic Statistical Authority (1955–2015).

**Results:**

During the crisis, mortality increased from 9.76/1000 in 2009 to 10.52/1000 in 2012 and to 11.16/1000 in 2015, driven by an increase in the number of deaths and a decrease in the estimated population. The annual increase of the expected mortality accelerated during the crisis; in contrast, age-adjusted mortality continued to decrease up to 2014 and increased in 2015. The subpopulations that seemed to be affected more during the crisis were the elderly (especially those over 70 years), women, and citizens in southern Greece. The common denominator of all these subgroups was older age. Mortality due to heart diseases continued to decline at an accelerated pace; due to neoplasia continued to increase at an accelerated pace; and stroke mortality reversed (from decline to increment).

**Conclusions:**

The increment of crude mortality during the financial crisis in Greece should be attributed to the increase in deaths, only in part due to the aging population, the reduction in births, and the increase in emigration that contracted the population.

## INTRODUCTION

In 2007 Greece experienced its ninth year of recession (2009–2017). Although Greece was never officially declared in default, several economic and social indices showed severe deterioration. The decrease in gross domestic product (GDP), increasing national debt, and high deficits were accompanied by an increase in unemployment and poverty.^1^ Inevitably, the financial crisis resulted in reforms in insurance and health policies. Among others, restructuring of the hospital sector (by closing hospitals or cutting their budgets), reductions in salaries of the health professionals, reductions of the extent of coverage by instituting or increasing user charges for health services, reductions in absolute public health expenditure, and changes in the pharmaceutical market (drug shortages and delayed reimbursements to pharmacies despite lower prices of the expanding generic medication market) were observed.^2^,^3^ According to data from the Hellenic Statistical Authority (ELSTAT), in the year 2005, 317 hospitals and medical centers were functioning in Greece, while in 2016 this number was reduced to 280.

Several articles have been published demonstrating changes between the periods before and during the financial crisis in several health indices.^3^ Greece saw an increase in suicide rate and mortality,^4^–^6^ deteriorating self-reported general health,^7^,^8^ decreasing access to health care and patients not receiving treatment due to lack of insurance,^7^,^9^ increases in admissions to public and decreases to private hospitals,^10^,^11^ and an HIV outbreak.^12^ On the other hand, after studying the changes of 30 health indicators during the period 1990–2011, an in-depth review on the impact of economic crisis and austerity in health concluded that “the evidence does not support the claim that there is a health crisis in Greece.”^13^

An increase in crude mortality has been observed in the years up to 2012, which was attributed to the aging population (67%) and austerity (33%). However, the age-adjusted mortality was declining.^14^ The publication of mortality data delay is approximately 2 years, and new data have become available. Greece faces a prolonged recession, and continuing cuts to health expenditure had probably affected health services further. A deeper investigation of the subpopulations mostly affected by these changes and the diseases contributing more to mortality changes was not performed herein or elsewhere. In this study we sought to explore these factors and extend the studied years to 2015.

## METHODS

Using data from the Hellenic Statistical Authority (ELSTAT), we calculated crude mortality for the entire population and for subgroups according to gender, cause of death (as reported in death certificates), age groups (0–19, 20–39, 40–55, 56–69, and 70 or older), and geographic regions (North and South Greece) for the period 2000–2015. Additional data (1955–1999) were incorporated to evaluate if changes in mortality occurred long before the study period. The study was divided in two periods: 2000–2009 (pre-crisis) and 2010–2015 (during the crisis). Although the financial crisis appeared in late 2008, its effects in the Greek population became evident in 2010, when cuts in salaries and public investments were imposed and unemployment rose above 10%. For this reason, the years 2010–2015 were selected as the financial crisis period. This study was approved by the Ethics Committee of the Alfa Institute of Biomedical Sciences, Athens, Greece. Acknowledgement of the source of the data taken from the Hellenic Statistical Authority (ELSTAT) is made.

The size of each year’s total population corresponds to the “estimated population,” which is based on the decennial Census of Population and is updated annually by the births, deaths, and movement of immigrants/emigrants occurring meanwhile. All death causes were categorized into 16 groups based on the relevant list provided by the ELSTAT. Those referring to the same organ system were merged into one category, whereas causes accounting for a very small number of deaths were omitted. Mortality trends of the six main causes of death were studied in detail.

It is known that the Greek population grew older throughout the study period. We hypothesized that if mortality was affected only by the age of the population, aging would have resulted in a continuous annual mortality increment. To test this hypothesis, the age distribution of the population in year 2001, when the census was performed, was selected as the reference population. To calculate mortality rates, the population was divided into 18 age subgroups, i.e. 0–4 years, 5–9 years etc., till 80–84 years and ≥85 years. The number of deaths and the number of people for every age subgroup in the year 2001 was recorded. Crude mortality (per 1000 population) for every subgroup was calculated. To calculate the expected mortality for the next years, we projected the estimated Greek population for every year to the mortality observed in the reference year 2001. A coefficient for every age subgroup was calculated by dividing the number of people in each subgroup by the total population in the year under study (for example, the estimated population in the subgroup 0–4 years in the year 2004 over the total population in 2004). This coefficient was multiplied by the appropriate crude mortality in each age subgroup of the year 2001. Expected mortality in the year under study was the sum of all products. Expected mortality was calculated for the years from 2002 till 2015 and compared to the observed one. Age-adjusted mortality was calculated according to the 2001 Greek population and expressed per 1000 population.

## RESULTS

### Demographic Changes 1955–2015

**Figure 1:**
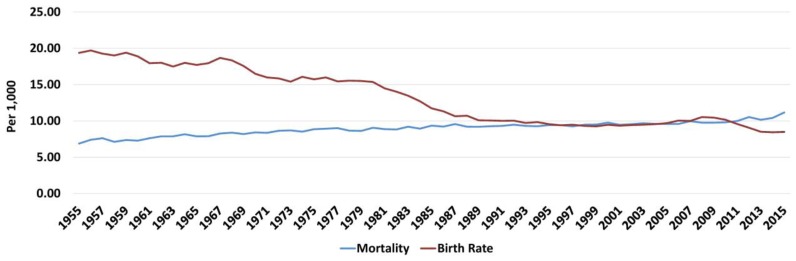
Mortality and Birth Rate in Greece (1955–2015). Data from the Hellenic Statistical Authority.

**Figure 2:**
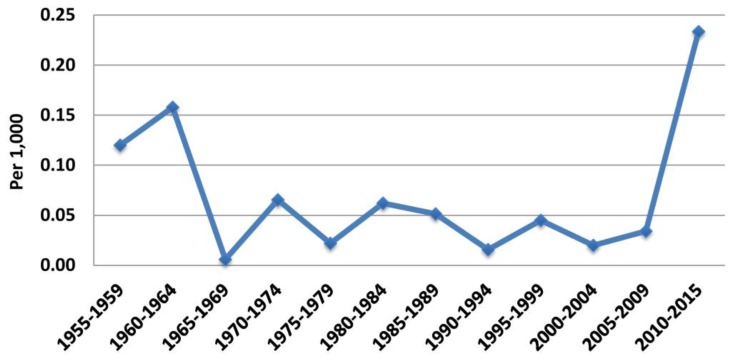
Mean 5-year Mortality Changes Rate, 1955–2015. Data from the Hellenic Statistical Authority.

**Figure 3:**
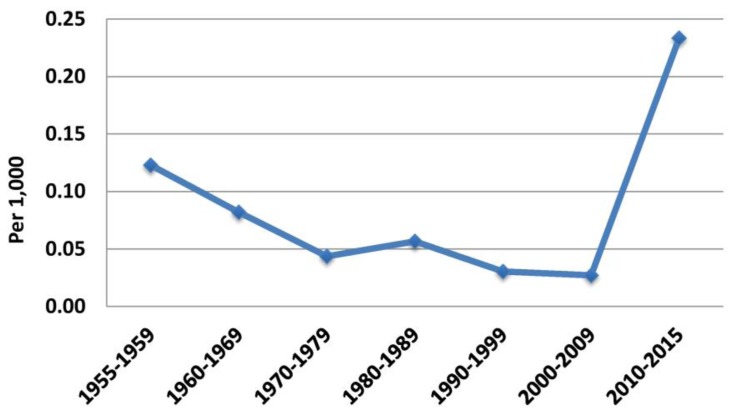
Mean 10-year Mortality Changes Rate, 1955–2015. Data from the Hellenic Statistical Authority.

ELSTAT has provided complete annual data for births and deaths in Greece since 1955. An almost continuous decrease in annual births (and birth rate) has been accompanied by an almost continuous increase in annual deaths (and mortality) from 1955 till 2015 (Figure 1). The first decade of the twenty-first century was an exception for births only (birth rate increased); mortality continued to increase. However, at the beginning of the second decade birth rate dropped significantly, while mortality continued to increase. Regarding birth rate, mean annual changes in each quinquennium (5-year period) and decade were negative, apart from the periods 1965–1969 and 2000–2009. Regarding mortality, the respective changes were always positive. However, although there was a decline in annual mortality increment rate from 1955 to 2009 (mean annual changes for each quinquennium and decade, from 0.12/1000 in 1955–1959 to 0.03/1000 in 2005–2009; Supplementary Table 1), this rate increased to 0.23/1000 population in the years 2010–2015 (Figures 2 and 3).

### Mortality in the Population in the Period 2000–2015

**Figure 4:**
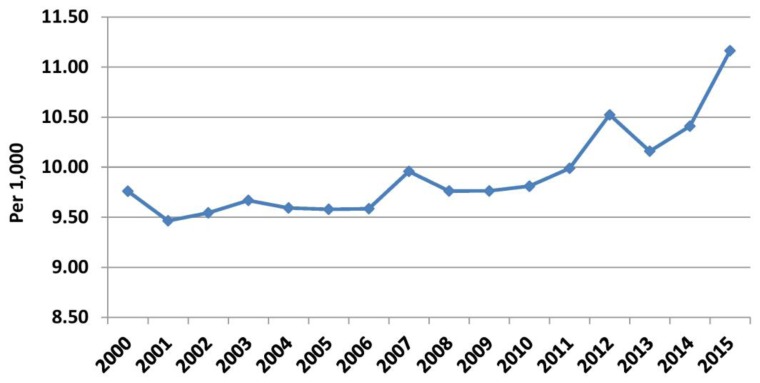
Mortality in Greece in the Period 2000–2015. Data from the Hellenic Statistical Authority.

During the pre-crisis period, mortality remained almost unchanged: 9.76/1000 in 2000, a sudden drop to 9.46/1000 in 2001, a sudden increase to 9.96 in 2007, and finally 9.76/1000 in 2009. During the crisis, mortality increased from 9.81/1000 in 2010 to 10.52/1000 in 2012 and to 11.16/1000 in 2015 (Figure 4). This increase in mortality should be attributed more to the increase in the number of deaths and to a lesser extent in the reduction of the estimated Greek population (due to the reduction of births and increase in emigration). Before the crisis, the mean annual increment in the total number of deaths was 349.56, which during the crisis increased to 2149.33 (increased by a factor of 6.15). The mean annual increase of the estimated Greek population was 35,457.56 before the crisis, while during the crisis the estimated population decreased annually by 39,454.50 (Supplementary Table 2).

**Figure 5:**
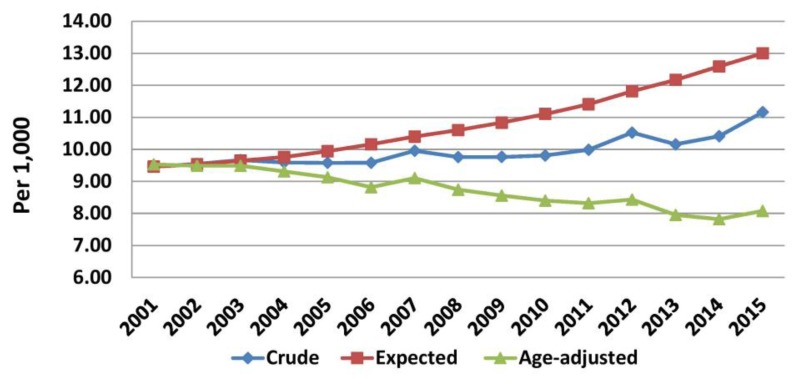
Observed, Projected, and Age-adjusted Mortality (2001–2015). Data from the Hellenic Statistical Authority.

Figure 5 shows the crude observed, expected, and age-adjusted mortality to the reference Greek population of 2001. The observed mortality was lower than the expected one despite the aging of the Greek population (Supplementary Table 3) throughout the study period. The annual increase of the expected mortality accelerated during the crisis (0.36/1000 versus 0.17/1000). In contrast, age-adjusted mortality continued to decrease throughout the study period. However, during the crisis the mean annual decrease was lower (0.08/1000) than that before (0.12/1000).

### Age Subgroups

In the general population (Supplementary Table 4), the increase in mortality can be attributed to the increase in mortality among people 70 years and older, and especially among those 80 years and older. Mortality dropped annually before and during the crisis in the age groups 0–19 (mean 0.004/1000 and 0.002/1000, respectively) and 20–39 (0.003/1000 and 0.013/1000, respectively); in both age groups the decrease should mainly be attributed to the decrease in the number of deaths. Mortality dropped annually before (mean 0.001/1000) and during the crisis (mean 0.005/1000) in the age groups 40–54; the mortality drop should be attributed to the increase of the population of this subgroup and to a lesser extent to the decrease in the number of deaths. Mortality decreased annually before the crisis (0.037/1000) and increased during the crisis (0.034/1000) in the age groups 55–69 and should be attributed to the increase in the number of deaths, as the population also increased. Mortality increased annually before (0.05/1000) and during the crisis (0.22/1000) in the age group 70 and older. This should be attributed to a relatively lower increase in new entries in the 70 and older population and primarily in an increase in deaths.

In specific age subgroups (Supplementary Table 5), mortality among people 0–19 years of age dropped annually in both periods (before 0.02/1000; and during the crisis 0.009/1000) due to the decrease in the number of deaths. Among those 20–39 and 40–54 years old the drop in mortality during the crisis should be primarily attributed to the decrease in the number of deaths; the decrease was more evident in the age group 20–39 (mean annual drop 0.0002/1000 before and 0.03/1000 during the crisis) than in the age group 40–54 (0.02/1000 and 0.04/1000, respectively). The mean annual drop before the crisis in the age group 55–69 (0.17/1000) reversed during the crisis (increase 0.06/1000), and should be attributed to the increase in the number of deaths. Finally, in the age group 70 and older, mortality increased annually during the crisis (0.69/1000) compared to a decreasing rate before (0.54/1000). This should be attributed to a relatively higher increase in new entries in the 70 and older population compared to the increase in deaths.

### Gender

**Figure 6:**
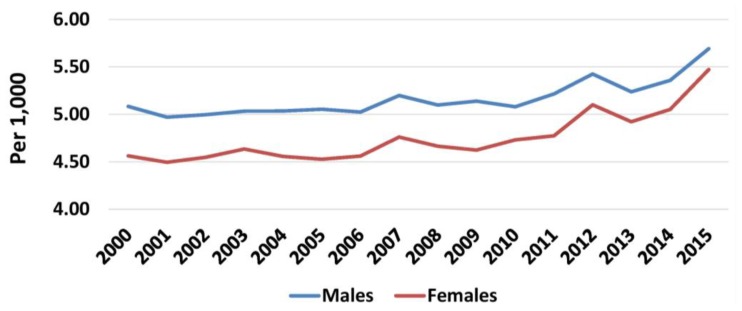
Mortality in Males and Females, 2000–2015. Data from the Hellenic Statistical Authority.

Mortality in the general population increased for both males (5.08/1000 in 2000 and 5.69/1000 in 2015) and females (4.56/1000 and 5.47/1000, respectively) during the crisis (Figure 6). Male deaths increased annually both before and during the crisis, but the rate of increase was higher during (797/year) than before the crisis (177/year). The male population began to decrease soon during the crisis (mean 31,284/year), while it was increasing before (15,755.9/year). Thus, male mortality in the general population increased annually in both periods, but at a higher rate during the crisis (0.09/1000 compared to 0.01/1000 before) mainly due to the reduction in the estimated population. On the other hand, female deaths increased sharply during the crisis (1352 more deaths per year) compared to before (172.6 per year), but changes in estimated female population were less marked (16,588.63 mean annual increase before, 8170 mean annual decrease during the crisis). Therefore, the higher mean annual increase in female mortality during the crisis (0.14/1000) compared to before (0.01/1000) should be attributed mainly to the increase in the number of deaths (Supplementary Table 6).

### Causes of Death

**Figure 7:**
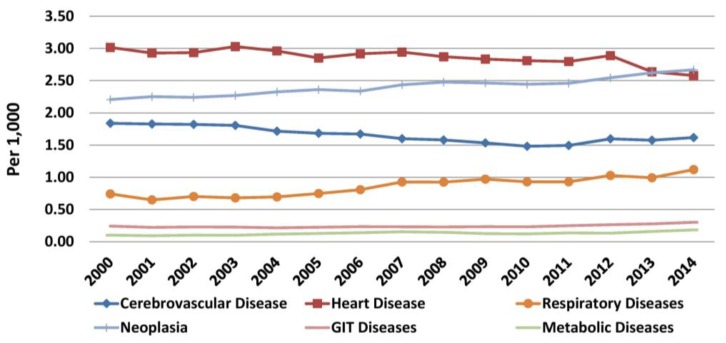
Main Causes for Mortality in Greece (2000–2014). Data from the Hellenic Statistical Authority.

Data for causes of death were available up to 2014. The main causes of death in Greece before and during the crisis remained unchanged: heart diseases, cancer, cerebrovascular disease, respiratory diseases, diseases of the gastrointestinal tract, and metabolic diseases (Figure 7). Heart diseases were the commonest cause of death throughout the study period, but mortality declined almost continuously (from 3.01/1000 in 2000 to 2.58/1000 in 2014). Moreover, the annual rate of decline during the crisis increased (0.05/1000) compared to the one before the crisis (0.02/1000), which should be primarily attributed to the decrease in deaths due to heart diseases. Cancer was the second most common cause of death and showed a steady increase in mortality (from 2.21/1000 in 2000 to 2.67/1000 in 2014). In fact, the rate of annual increase in patients with cancer increased during the crisis (0.04/1000 compared to 0.03/1000 before). During the last year of the study, cancer became the number one cause of death in Greece. Since the annual increase in deaths during the crisis (mean 362) was lower than before (396.7), the increase should be mainly attributed to the decrease of the estimated population.

In cerebrovascular disease, the continuous decline in mortality before the crisis (mean 0.03/1000 per year) was reversed during the crisis (0.02/1000). Thus, mortality was 1.84/1000 in 2000, dropped to 1.48/1000 in 2010, and increased to 1.62/1000 in 2014 and should be mainly attributed to the increase of deaths. Mortality due to respiratory diseases increased at the same rate in both periods (0.03/1000). Mortality due to diseases of the digestive system was decreasing slightly before the crisis (0.001/1000 per year) but began to increase during (0.01/1000 per year). Finally, mortality due to metabolic diseases increased both before (mean annual 0.003/1000) and during the crisis (0.01/1000).

**Figure 8:**
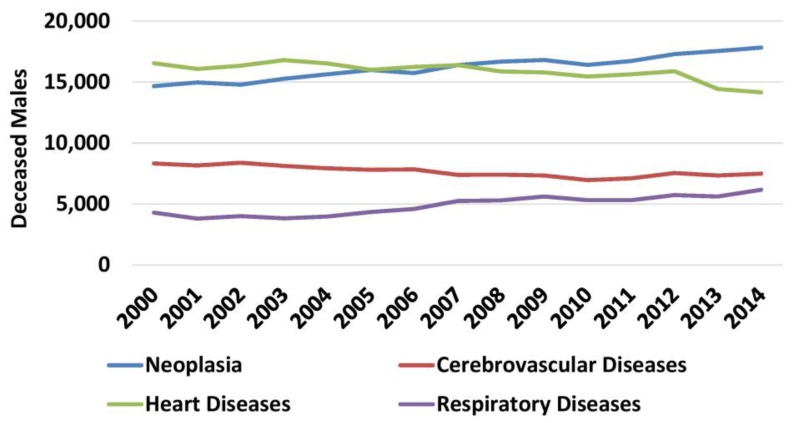
Mortality Causes among Males 2000–2014. Data from the Hellenic Statistical Authority.

**Figure 9:**
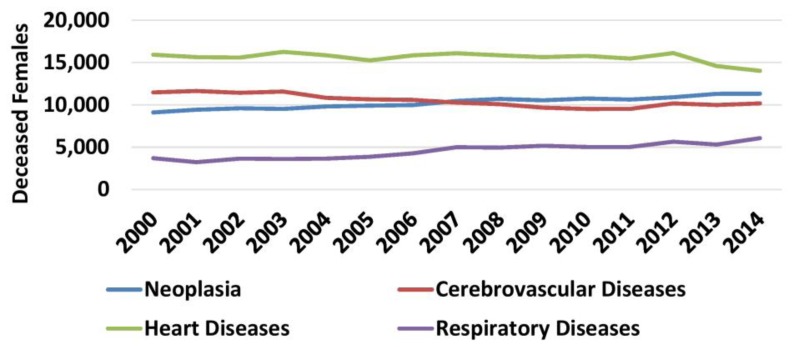
Mortality Causes among Females 2000–2014. Data from the Hellenic Statistical Authority.

For the four main causes of death, mortality was studied according to gender. In general, in both genders annual changes in mortality throughout the study period were similar to the general population. However, cancer became the first cause of death among males in 2009, surpassing heart diseases (Figure 8). Similarly, cancer became the second cause of death among females in 2009 surpassing cerebrovascular diseases (Figure 9, Supplementary Table 7).

### Regions

**Figure 10:**
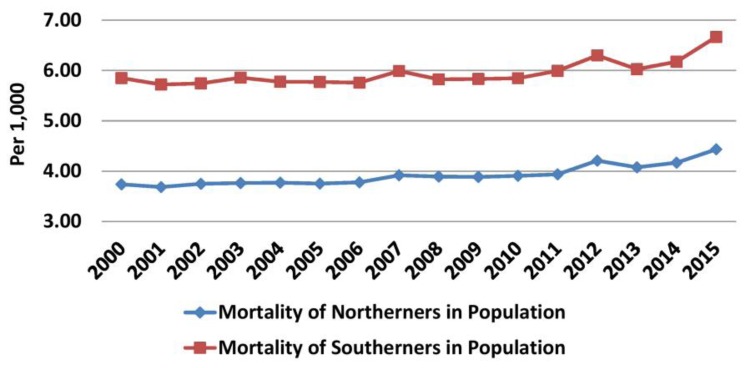
Mortality in South and North Greece in Population. Data from the Hellenic Statistical Authority.

**Figure 11:**
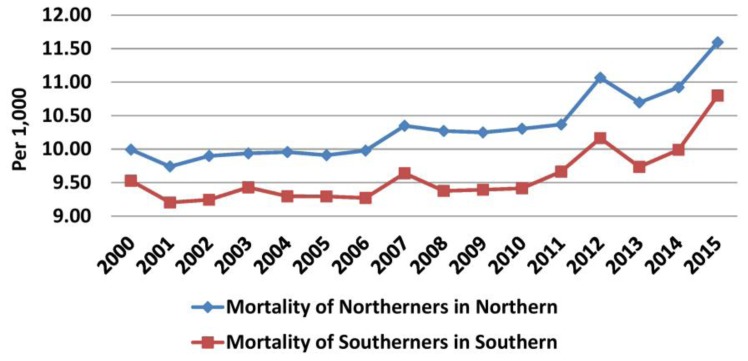
Mortality in South and North Greece in Subpopulations. Data from the Hellenic Statistical Authority.

Increases in the number of deaths and the rate of annual increase (260.6 before versus 839 during the crisis) were observed in northern Greece in both periods. The population in this region increased annually until 2011, but it started to decline thereafter. Thus, mortality was increasing annually before the crisis (from 3.74 in 2000 to 3.88/1000 in 2009, mean annual increase 0.02/1000), but the rate of increase became higher during the crisis (3.91/1000 in 2010 to 4.43/1000 in 2015, mean annual increase 0.05/1000) (Figures 10 and 11, Supplementary Table 8). In southern Greece the increase in the number of deaths was lower than in northern Greece before the crisis (101.4/year), and in combination with the higher increase in the population this resulted in a small annual decrease in mortality (0.002/1000 per year). However, during the crisis the rate of increase in deaths (1282.33/year) and the decline in the population (30,486.5/year) were more evident. Thus, mortality increased from 5.85/1000 in 2010 to 6.67/1000 in 2015 (mean annual increment 0.14/1000) (Supplementary Table 8).

Regarding specific districts, four different patterns were observed (Supplementary Table 9). In Attica, Western Macedonia, Central Macedonia, and South Aegean Islands mortality increased in both periods, but with higher rate during the crisis. In Eastern Macedonia and Thrace and in Thessaly it increased in both periods, but decelerated during the crisis. In Epirus, Ionian Islands, Western Greece, Central Greece, and Crete it decreased before and increased during the crisis. In Peloponnese and North Aegean it decreased in both periods, but with a lower rate during the crisis.

## DISCUSSION

According to the data provided by ELSTAT, crude mortality increases continuously in Greece from 1955. Although the rate of this increase was declining up to 2009, it suddenly accelerated during the period 2010–2015 to reach levels almost twice as high as that of the period 1955–1959. However, life expectancy (i.e. population aging) continued to increase.^15^ Along with the increase in the number of deaths in the period 2010–2015, a reduction of births and an increase in emigration were observed. Thus, these parallel phenomena led to the increase in crude mortality during the economic crisis. The increase in mortality should be attributed mainly to the increase in the number of deaths, and to a lesser extent to the decrease of the population. This sudden increase cannot be explained by the population aging.

The increase in crude mortality was not observed across all Greek subpopulations. In fact, the mortality increment observed before the crisis in several subgroups either decelerated or even reversed during the crisis. Thus, the subpopulations that seemed to be affected more during the crisis were specific age groups (especially those over 70 years), women, and citizens in South Greece. The common denominator of all these subgroups was older age. The mortality curve in people older than 70 years was almost identical to the curve of the general population; people 80 years or older showed the highest increase in mortality. Women and southerners have a greater life expectancy and were likely older by the time of death.^16^ Similar findings have been reported for elderly females in Spain and Russia, but not in South Korea.^17^–^19^

Mortality due to heart diseases continued to decrease during the crisis, despite reports showing an increase in arrhythmias, myocardial infarction, and heart failure during the crisis.^20^,^21^ This probably denotes the progress in prevention and treatment of heart diseases,^22^,^23^ and is consistent with similar decreases observed in European countries and USA.^24^,^25^ On the other hand, the decreasing mortality from cerebrovascular disease was reversed in 2012 and 2013 and affected both genders. Mortality from the remaining main causes of death, i.e. neoplasia, respiratory, and digestive and metabolic diseases, continued to increase during the crisis. Age-adjusted mortality was shown to decrease in heart diseases, neoplasia, and road traffic accidents.^23^,^26^,^27^

Greece suffers a severe demographic problem, with mortality surpassing the birth rate; emigration poses additional threats. The population ages, a phenomenon common to most European countries.^28^ However, beginning in 2011 a continuous decline in the Greek population has been observed, an unprecedented phenomenon in its recent history following the Second World War. If the current condition continues, the Greek population will continue to contract, and it is estimated to be as low as 8.3 million by 2050.^29^

Whether it is the economic crisis per se or other causes that conferred more to the increase in mortality was not evaluated in this study. Previous studies reported conflicting results depending on the methodology they used (panel data analysis, time series analysis, etc.), the indicator used as a measurement of recession (GDP, unemployment, GDP per capita), the duration or the type of the recession (normal fluctuation or severe crisis), the pre-existing levels of vulnerability (public infrastructure, social safety nets, access to effective healthcare, and education), the quality of responses to a crisis (cuts in public spending and health expenditures), and the population(s) under study (developed or developing countries, individual or aggregate relationships).^30^–^36^ Thus, Stuckler et al. concluded that a rise in unemployment (used as an example because it is considered the most credible marker) during economic cycles did not affect all cause-adjusted mortality in 26 countries of the European Union for the period 1970–2007.^35^ However, they reported that increases in unemployment were associated with increased suicide, alcohol abuse, and homicide mortality.^35^ On the other hand, in two different papers, Neumayer^37^ and Ruhm^38^ concluded that higher unemployment was associated with lower mortality overall, also for specific causes, including cardiovascular diseases. They both accepted that the positive impact of recessions (healthier lifestyle in the population, reduced industrial and injury-related deaths, better immunity)^39^,^40^ may more than compensate for the negative health effects on the unemployed. Finally, Brenner and Mooney concluded that unemployment and population health have an inverse association.^41^

The key points from this study are as follows:

- With ups and downs, during the pre-crisis period mortality remained almost unchanged.
- During the economic crisis, mortality increased, driven by an increase in the number of deaths and a decrease in the estimated population; in contrast, age-adjusted mortality continued to decrease.
- The subpopulations that seemed to be affected more during the crisis were the elderly (especially those over 70 years), women, and citizens in South Greece. The common denominator of all these subgroups was older age.
- Mortality due to heart diseases continued to decline at an accelerated pace; mortality due to neoplasia continued to increase at an accelerated pace, and that due to stroke was reversed (from decline to increment).

## CONCLUSION

Mortality in Greece increased in the first years of economic crisis, mainly due to the increase in the number of deaths and could be only in part attributed to the aging of the population. In addition, specific subgroups, mainly women and citizens of southern Greece seem to be affected more. It is difficult to calculate the contribution of the economic crisis itself to these observations. However, as the crisis continues and in some cases evolves, it is possible that only its early effects are apparent so far; its full scale of consequences in Greece will probably become apparent in the years to come.

## Supplementary Data


