# Supplementary material for: Trends of Mortality in Greece Prior to and During Its Current Financial Crisis (2009–2015)
Source: Rambam Maimonides Med J. 2019 Jul 18;10(3):e0015. doi: 10.5041/RMMJ.10368 (PMC6649780; doi:10.5041/RMMJ.10368)
Supplement: Supplementary file 1 [file rmmj-10-3-e0015-sup.pdf]

This appendix has been provided by the authors to give readers additional background reading and information

Supplement to: Trends of Mortality in Greece Prior to and During its Current Financial Crisis (2009-2015)

Vardakas KZ, Kyriakidou M, Apiranthiti KN, Almpani SE, Heliou D, Stratigopoulou D, Giourmetaki E, Lamprou D, Binou G, Mpaltzoglou E, Falagas ME. Trends of Mortality in Greece Prior to and During its Current Financial Crisis (2009-2015). Rambam Maimonides Med J 2019;10 (3):e0015. doi:10.5041/RMMJ.10368

Supplementary Table 1. Mean Annual Changes in Mortality and Birth Rate by Quinquennium and Decade (1955-2015).

Quinquennium	Mean Mortality Changes	Mean Birth Rate Changes	Decade	Mean Mortality Changes	Mean Birth Rate Changes
1955-1959	0.12	0.01	1955-1959	0.12	0.01
1960-1964	0.16	-0.28			
1965-1969	0.01	0.09	1960-1969	0.08	-0.18
1970-1974	0.07	-0.3			
1975-1979	0.02	-0.12	1970-1979	0.04	-0.21
1980-1984	0.06	-0.56			
1985-1989	0.05	-0.53	1980-1989	0.06	-0.54
1990-1994	0.02	-0.05			
1995-1999	0.04	-0.12	1990-1999	0.03	-0.08
2000-2004	0.02	0.06			
2005-2009	0.03	0.18	2000-2009	0.03	0.12
2010-2015	0.23	-0.33	2010-2015	0.23	-0.33

Supplementary Table 2. Estimated Population, Deaths, and Mortality (per 1000 Population) in Greece, 2000-2015.

Year	2000	2001	2002	2003	2004	2005	2006	2007	2008	2009	2010	2011	2012	2013	2014	2015
Deaths (Total)	105,170	102,559	103,915	105,529	10,4942	10,5091	105,476	109,895	107,979	108,316	109,084	111,099	116,668	111,794	113,740	121,212
Annual Change		-2611	1356	1614	-587	149	385	4419	-1916	337	768	2015	5569	-4874	1946	7472
Mean Change										349.56*						2149.333**
Population (Total)	10,775,627	10,835,989	10,888,274	10,915,770	10,940,369	10,969,912	11,004,716	11,036,008	11,060,937	11,094,745	11,119,289	11,12,3392	11,08,6406	11,003,615	10,926,807	10,858,018
Annual Change		60,362	52,285	27,496	24,599	29,543	34,804	31,292	24,929	33,808	24,544	4,103	-36,986	-82,791	-76,808	-68,789
Mortality per 1000	9.76	9.46	9.54	9.67	9.59	9.58	9.58	9.96	9.76	9.76	9.81	9.99	10.52	10.16	10.41	11.16
Annual Change		-0.3	0.08	0.12	-0.08	-0.01	0	0.37	-0.2	0	0.05	0.18	0.54	-0.36	0.25	0.75
Mean Change										0.0003*						0.23**

* Mean annual difference for the period 2000-2009

** Mean annual difference for the period 2010-2015

Supplementary Table 3. Crude, Projected, and Age-adjusted Mortality (per 1000 Population) in Greece, 2001-2015.

Year	2001	2002	2003	2004	2005	2006	2007	2008	2009	2010	2011	2012	2013	2014	2015
Crude Mortality	9.46	9.54	9.67	9.59	9.58	9.58	9.96	9.76	9.76	9.81	9.99	10.52	10.16	10.41	11.16
Annual Change		0.08	0.12	-0.08	-0.01	0	0.37	-0.2	0	0.05	0.18	0.54	-0.36	0.25	0.75
Mean Change									0.04				0.1		0.23
Projected Mortality	9.46	9.53	9.65	9.76	9.94	10.16	10.4	10.6	10.83	11.1	11.41	11.81	12.17	12.59	13
Annual Change		0.07	0.12	0.11	0.18	0.22	0.24	0.21	0.23	0.27	0.31	0.4	0.36	0.42	0.41
Mean Change									0.17				0.33		0.36
Age-adjusted Mortality	9.53	9.49	9.49	9.31	9.13	8.82	9.1	8.74	8.56	8.4	8.32	8.43	7.95	7.82	8.08
Annual Change		-0.04	0	-0.18	-0.18	-0.31	0.28	-0.36	-0.18	-0.16	-0.08	0.11	-0.48	-0.13	0.26
Mean Change									-0.12						-0.08
Crude/ Projected Mortality	1	1	1	0.98	0.96	0.94	0.96	0.92	0.9	0.88	0.88	0.89	0.83	0.83	0.86

Supplementary Table 4. Mortality (per 1000 Population) in Age Subgroups in Greece, 2000-2015.

Year	2000	2001	2002	2003	2004	2005	2006	2007	2008	2009	2010	2011	2012	2013	2014	2015
Deaths 1-19	1,149	1,103	1,052	941	944	930	853	838	712	805	856	751	649	639	667	655
Deaths 20-39	2,274	2,647	2,447	2,433	2,600	2,722	2,532	2,654	2,395	2,526	2,264	2,094	2,010	1,710	1,618	1,598
Deaths 40-54	5,550	5,469	5,356	5,399	5,425	5,407	5,499	5,712	5,531	5,589	5,451	5,538	5,474	5,331	5,275	5,153
Deaths 55-69	18,085	17,445	16,900	16,832	16,583	15,762	15,514	15,673	15,268	14,858	14,799	15,270	16,031	15,989	16,107	16,739
Deaths 70+	77,662	75,895	78,160	79,924	79,390	80,270	81,078	85,018	84,073	84,538	85,714	87,446	92,504	88,125	90,073	97,067
Mortality 1-19 per 1000	0.107	0.102	0.097	0.086	0.086	0.085	0.078	0.076	0.064	0.073	0.077	0.068	0.059	0.058	0.061	0.06
Annual Difference		-0.005	-0.005	-0.01	0	-0.002	-0.007	-0.002	-0.012	0.008	0.004	-0.009	-0.009	0	0.003	-0.001
Mean Change										-0.005*						-0.002**
Mortality 20-39 per 1000	0.211	0.244	0.225	0.223	0.238	0.248	0.23	0.24	0.217	0.228	0.204	0.188	0.181	0.155	0.148	0.147
Annual Difference		0.03	-0.02	0	0.01	0.01	-0.02	0.01	-0.02	0.01	-0.02	-0.02	-0.01	-0.03	-0.01	0
Mean Change										0.002*						-0.01**
Mortality 40-54	0.515	0.505	0.492	0.495	0.496	0.493	0.5	0.518	0.5	0.504	0.49	0.498	0.494	0.484	0.483	0.475
Annual Difference		-0.01	-0.01	0	0	0	0.01	0.02	-0.02	0	-0.01	0.01	0	-0.01	0	-0.01
Mean Change										-0.001*						-0.005**
Mortality 55-69	1.678	1.610	1.552	1.542	1.516	1.437	1.410	1.420	1.380	1.339	1.331	1.373	1.446	1.453	1.474	1.542
Annual Difference		-0.07	-0.06	-0.01	-0.03	-0.08	-0.03	0.01	-0.04	-0.04	-0.01	0.04	0.07	0.01	0.02	0.07
Mean Change										-0.038						0.034**
Mortality 70+	7.207	7.004	7.178	7.322	7.257	7.317	7.368	7.704	7.601	7.620*	7.709	7.861	8.344	8.009	8.243	8.940
Annual Difference		-0.2	0.17	0.14	-0.07	0.06	0.05	0.34	-0.1	0.02	0.09	0.15	0.48	-0.34	0.23	0.7
Mean Change										0.046						0.22

* Mean annual difference for the period 2000-2009

** Mean annual difference for the period 2010-2015

Supplementary Table 5. Mortality (per 1000 in the Subgroup) in Age Subgroups in Greece, 2000-2015.

Year	2001	2002	2003	2004	2005	2006	2007	2008	2009	2010	2011	2012	2013	2014	2015
Population 1-19	2,258,027	2,407,640	2,363,241	2,323,788	2,294,236	2,271,844	2,252,213	2,239,006	2,224,700	2,214,295	2,199,813	2,183,632	2,161,800	2,144,124	2,115,564
Mortality 1-19 per 1000	0.49	0.44	0.4	0.41	0.41	0.38	0.37	0.32	0.36	0.39	0.34	0.3	0.3	0.31	0.31
Annual Change		-0.05	-0.04	0.01	0	-0.03	0	-0.05	0.04	0.02	-0.05	-0.04	0	0.02	0
Mean Change									-0.016*						-0.009**
Population 20-39	33,47,511	32,98,178	3,311,837	3,320,331	3,318,701	3,308,654	3,281,333	3237428	3,200,086	3,153,136	3,095,705	3,021,703	2,930,141	2,836,295	2,751,986
Mortality 20-39 per 1000	0.79	0.74	0.73	0.78	0.82	0.77	0.81	0.74	0.79	0.72	0.68	0.67	0.58	0.57	0.58
Annual Change		-0.05	-0.01	0.05	0.04	-0.05	0.04	-0.07	0.05	-0.07	-0.04	-0.01	-0.08	-0.01	0.01
Mean Change									-0.0002*						-0.035**
Population 40-54	2,111,642	2,110,451	2,128,110	2,146,566	2,168,713	2,195,542	2,227,858	2,267,805	2,309,857	2,345,279	2,370,592	2,380,772	2,381,188	2,376,703	2,374,411
Mortality 40-54 per 1000	2.59	2.54	2.54	2.53	2.49	2.5	2.56	2.44	2.42	2.32	2.34	2.3	2.24	2.22	2.17
Annual Change		-0.05	0	-0.01	-0.03	0.01	0.06	-0.12	-0.02	-0.1	0.01	-0.04	-0.06	-0.02	-0.05
Mean Change									-0.021*						-0.042**
Population 55-69	1,824,826	1,767,371	1,772,811	1,774,883	1,776,419	1,780,183	1,787,593	1,794,932	1,803,253	1,818,091	1,844,557	1,873,730	1,900,224	1,928,499	1,953,000
Mortality 55-69 per 1000	9.56	9.56	9.49	9.34	8.87	8.71	8.77	8.51	8.24	8.14	8.28	8.56	8.41	8.35	8.57
Annual Change		0	-0.07	-0.15	-0.47	-0.16	0.05	-0.26	-0.27	-0.1	0.14	0.28	-0.14	-0.06	0.22
Mean Change									-0.165*						0.055**
Population 70+	1,293,983	1,304,634	1,339,771	1,374,801	1,411,843	1,448,493	1,487,011	1,521,766	1,556,849	1,588,488	1,612,725	1,626,569	1,630,262	1,641,485	1,660,765
Mortality 70+ per 1000	58.65	59.91	59.65	57.75	56.85	55.97	57.17	55.25	54.3	53.96	54.22	56.87	54.06	54.87	58.45
Annual Change		1.26	-0.25	-1.91	-0.89	-0.88	1.2	-1.93	-0.95	-0.34	0.26	2.65	-2.81	0.82	3.57
Mean Change									-0.544						0.691

* Mean annual difference for the period 2000-2009

** Mean annual difference for the period 2010-2015

Supplementary Table 6. Mortality (per 1000 Population and in the Subgroup) in Males and Females in Greece, 2000-2015.

Year	2000	2001	2002	2003	2004	2005	2006	2007	2008	2009	2010	2011	2012	2013	2014	2015
Male Deaths	55,422	53,853	54,406	54,942	55,084	55,435	55,290	57,366	56,390	57,015	56,480	57,999	60,135	57,630	58,546	61,798
Male Population		5,330,048	5,381,045	5,394,780	5,406,076	5,418,462	5,433,391	5,442,245	5447,947	5,456,095	5,460,865	5,453,444	5,423,884	5,366,251	5,313,239	5,268,390
Female Deaths	49748	48,706	49,509	50,587	49,858	49,656	50,186	52,529	51,589	51,301	52,604	53,100	56,533	54,164	55,194	59,414
Female Population		5,505,941	5,507,229	5,520,990	5,534,293	5,551,450	5,571,325	5,593,763	5,612,990	5,638,650	5,658,424	5,669,948	5,662,522	5,637,364	5,613,568	5,589,628
Male Mortality per 1000 Males		10.1	10.11	10.18	10.19	10.23	10.18	10.54	10.35	10.45	10.34	10.64	11.09	10.74	11.02	11.73
Annual Change			0.01	0.07	0	0.04	-0.05	0.36	-0.19	0.1	-0.11	0.29	0.45	-0.35	0.28	0.71
Mean Change										0.04*						0.29**
Male Mortality in Population	5.08	4.97	5	5.03	5.03	5.05	5.02	5.2	5.1	5.14	5.08	5.21	5.42	5.24	5.36	5.69
Annual Change		-0.11	0.03	0.04	0	0.02	-0.03	0.17	-0.1	0.04	-0.06	0.13	0.21	-0.19	0.12	0.33
Mean Change										0.01*						0.09**
Female Mortality per 1000 Females		8.85	8.99	9.16	9.01	8.94	9.01	9.39	9.19	9.1	9.3	9.37	9.98	9.61	9.83	10.63
Annual Change			0.14	0.17	-0.15	-0.06	0.06	0.38	-0.2	-0.09	0.2	0.07	0.62	-0.38	0.22	0.8
Mean Change										0.03*						0.26**
Female Mortality in Population	4.56	4.49	4.55	4.63	4.56	4.53	4.56	4.76	4.66	4.62	4.73	4.77	5.1	4.92	5.05	5.47
Annual Change		-0.07	0.05	0.09	-0.08	-0.03	0.03	0.2	-0.1	-0.04	0.11	0.04	0.33	-0.18	0.13	0.42
Mean Change										0.01*						0.14**

* Mean annual difference for the period 2000-2009

** Mean annual difference for the period 2010-2015

Supplementary Table 7. Mortality (per 1000 Population) According to the Main Causes of Death in Greece, 2000-2014 (continued next page).

Year	2000	2001	2002	2003	2004	2005	2006	2007	2008	2009	2010	2011	2012	2013	2014
Deaths – Heart Diseases	32,487	31,728	31,944	33,069	32,391	31,275	32,108	32,484	31,744	31,447	31,234	31,114	32,012	29,014	28,182
Annual Change		-759	216	1,125	-678	-1,116	833	376	-740	-297	-213	-120	898	-2,998	-832
Mean Change										-115.6*					-653**
Deaths - Cancer	23,775	24,408	24,386	24,779	25,448	25,896	25,718	26871	27,386	27,345	27,177	27,357	28,201	28,857	29,155
Annual Change		633	-22	393	669	448	-178	1153	515	-41	-168	180	844	656	298
Mean Change										396.7*					362**
Deaths - CVD	19,796	19,787	19,819	19,694	18,759	18,463	18,405	17,655	17,470	17,006	16,475	16,627	17,716	17,328	17,677
Annual Change		-9	32	-125	-935	-296	-58	-750	-185	-464	-531	152	1089	-388	349
Mean Change										-310*					134.2**
Deaths - Respiratory Diseases	7,994	7,023	7,650	7,430	7,611	8,209	8,874	10,229	10,239	10,770	10,341	10,335	11,384	10,924	12,231
Annual Change		-971	627	-220	181	598	665	1,355	10	531	-429	-6	1049	-460	1,307
Mean Change										308.4*					292.2**
Deaths - GIT Diseases	2,614	2,414	2,502	2,470	2,358	2,452	2,561	2,554	2,534	2,585	2,573	2,772	2,918	3,049	3,294
Annual Change		-200	88	-32	-112	94	109	-7	-20	51	-12	199	146	131	245
Mean Change										-3.22*					141.8**
Deaths - Metabolic Diseases	1,107	1,002	1,115	1,096	1,286	1,403	1,535	1,694	1,605	1,404	1,360	1,513	1,471	1,759	2,009
Annual Change		-105	113	-19	190	117	132	159	-89	-201	-44	153	-42	288	250
Mean Change										33*				88.75**	121
Mortality - Heart Disease	3.01	2.93	2.93	3.03	2.96	2.85	2.92	2.94	2.87	2.83	2.81	2.8	2.89	2.64	2.58
Annual Change		-0.09	0.01	0.1	-0.07	-0.11	0.07	0.03	-0.07	-0.04	-0.03	-0.01	0.09	-0.25	-0.06
Mean Change										-0.02*					-0.05**
Mortality - Cancer	2.21	2.25	2.24	2.27	2.33	2.36	2.34	2.43	2.48	2.46	2.44	2.46	2.54	2.62	2.67
Annual Change		0.05	-0.01	0.03	0.06	0.03	-0.02	0.1	0.04	-0.01	-0.02	0.02	0.08	0.08	0.05
Mean Change										0.03*					0.04**
Mortality - CVD	1.84	1.83	1.82	1.8	1.71	1.68	1.67	1.6	1.58	1.53	1.48	1.49	1.6	1.57	1.62
Annual Change		-0.01	-0.01	-0.02	-0.09	-0.03	-0.01	-0.07	-0.02	-0.05	-0.05	0.01	0.1	-0.02	0.04
Mean Change										-0.03*					0.02**
Mortality - Respiratory Diseases	0.74	0.65	0.7	0.68	0.7	0.75	0.81	0.93	0.93	0.97	0.93	0.93	1.03	0.99	1.12
Annual Change		-0.09	0.05	-0.02	0.02	0.05	0.06	0.12	0	0.05	-0.04	0	0.1	-0.03	0.13
Mean Change										0.03*					0.03**
Mortality - GIT Diseases	0.24	0.22	0.23	0.23	0.22	0.22	0.23	0.23	0.23	0.23	0.23	0.25	0.26	0.28	0.3

Year	2000	2001	2002	2003	2004	2005	2006	2007	2008	2009	2010	2011	2012	2013	2014
Annual Change		-0.02	0.01	0	-0.01	0.01	0.01	0	0	0	0	0.02	0.01	0.01	0.02
Mean Change										-0.001*					0.01**
Mortality - Metabolic Diseases	0.1	0.09	0.1	0.1	0.12	0.13	0.14	0.15	0.15	0.13	0.12	0.14	0.13	0.16	0.18
Annual Change		-0.01	0.01	0	0.02	0.01	0.01	0.01	-0.01	-0.02	0	0.01	0	0.03	0.02
Mean Change										0.003*				0.008**	0.01

* Mean annual difference for the period 2000-2009

** Mean annual difference for the period 2010-2014

Abbreviations: CVD cerebrovascular disease, GIT gastrointestinal tract

Supplementary Table 8. Mortality (per 1000 Population and in the Subgroup) in Citizens of North and South Greece, 2000-2015.

Year	2000	2001	2002	2003	2004	2005	2006	2007	2008	2009	2010	2011	2012	2013	2014	2015
North Greece Population	4,077,621	4,098,903	4,123,025	4,134,049	4,143,525	4,154,081	4,166,618	4,179,418	4,190,179	4,204,729	4,216,738	4,223,167	4,215,701	4,192,795	4,171,678	4,150,921
Annual Change		21,282	24,122	11,024	9,476	10,556	12537	12,800	10,761	14,550	12,009	6,429	-7,466	-22,906	-21,117	-20,757
Mean Change										14,123.11*						-8968**
Deaths in North Greece	40,748	39,922	40,806	41,081	41,253	41,161	41,576	43,253	43,036	43,093	43,449	43,787	46,649	44,853	45,564	48129
Annual Change		-826	884	275	172	-92	415	1,677	-217	57	356	338	2,862	-1,796	711	2565
Mean Change										260.56*						839.3333**
Mortality of Northerners in Population	3.74	3.68	3.75	3.76	3.77	3.75	3.78	3.92	3.89	3.88	3.91	3.94	4.21	4.08	4.17	4.43
Annual Change		-0.05	0.06	0.02	0.01	-0.02	0.03	0.14	-0.03	-0.01	0.02	0.03	0.27	-0.13	0.09	0.26
Mean Change										0.02*						0.09**
Mortality of Northerners in Northerners	9.99	9.74	9.9	9.94	9.96	9.91	9.98	10.35	10.27	10.25	10.3	10.37	11.07	10.7	10.92	11.59
Annual Change		-0.25	0.16	0.04	0.02	-0.05	0.07	0.37	-0.08	-0.02	0.06	0.06	0.7	-0.37	0.22	0.67
Mean Change										0.03*						0.22**
South Greece Population	6,698,006	6,737,086	6,765,249	6,781,721	6,796,844	6,815,831	6,838,098	6,856,590	6,870,758	6,890,016	6,902,551	6,900,225	6,870,705	6,810,820	6,755,129	6,707,097
Annual Change		39,080	28,163	16,472	15,123	18,987	22,267	18,492	14,168	19,258	12,535	-2,326	-29,520	-59,885	-55,691	-48,032
Mean Change										21,334.44*						-30,486.5**
Deaths in South Greece	63,814	62,006	62,533	63,944	63,186	63,345	63,392	66,096	64,432	64,727	64,998	66,689	69,831	66,306	67,476	72421
Annual Change		-1,808	527	1,411	-758	159	47	2704	-1,664	295	271	1691	3,142	-3,525	1,170	4,945
Mean Change										101.4444*						1,282.333**
Mortality of Southerners in Population	5.85	5.72	5.74	5.86	5.78	5.77	5.76	5.99	5.83	5.83	5.85	6	6.3	6.03	6.18	6.67
Annual Change		-0.13	0.02	0.11	-0.08	0	-0.01	0.23	-0.16	0.01	0.01	0.15	0.3	-0.27	0.15	0.49
Mean Change										-0.002*						0.14**
Mortality of Southerners in Southerners	9.53	9.2	9.24	9.43	9.3	9.29	9.27	9.64	9.38	9.39	9.42	9.66	10.16	9.74	9.99	10.8
Annual Change		-0.32	0.04	0.19	-0.13	0	-0.02	0.37	-0.26	0.02	0.02	0.25	0.5	-0.43	0.25	0.81
Mean Change										-0.01*						0.23**

* Mean annual difference for the period 2000-2009

** Mean annual difference for the period 2010-2015

Table 9. Mortality (per 1000 Population) in Citizens of Specific Districts in Greece, 2000-2015 (continued next page).

Year	2000	2001	2002	2003	2004	2005	2006	2007	2008	2009	2010	2011	2012	2013	2014	2015
Attica Deaths	34,086	33,079	33,410	34,165	34,182	34,234	33,959	35,880	34,858	35,443	35,564	36,472	37,917	36,881	37,352	40,199
Mortality	3.13	3.05	3.07	3.13	3.12	3.12	3.09	3.25	3.15	3.19	3.2	3.28	3.42	3.35	3.42	3.7
Annual Change		-0.07	0.02	0.06	-0.01	0	-0.03	0.17	-0.1	0.04	0	0.08	0.14	-0.07	0.07	0.28
Mean Change										0.01*						0.08**
Ionian Islands Deaths	2,463	2,216	2,482	2,452	2,372	2,285	2,410	2,346	2,506	2,393	2,243	2,266	2,498	2,379	2,425	2,700
Mortality	0.23	0.2	0.23	0.22	0.22	0.21	0.22	0.21	0.23	0.22	0.2	0.2	0.23	0.22	0.22	0.25
Annual Change		-0.02	0.02	0	-0.01	-0.01	0.01	-0.01	0.01	-0.01	-0.01	0	0.02	-0.01	0	0.03
Mean Change										-0.001*						0.005**
Western Greece Deaths	7,254	7,115	6,825	7,240	7,172	7,315	7,266	7,411	7,320	7,373	7,341	7,386	7,706	7,367	7,314	7,827
Mortality	0.67	0.66	0.63	0.66	0.66	0.67	0.66	0.67	0.66	0.66	0.66	0.66	0.7	0.67	0.67	0.72
Annual Change		-0.01	-0.03	0.04	-0.01	0.01	-0.01	0.01	-0.01	0	0	0	0.03	-0.03	0	0.05
Mean Change										-0.0001*						0.008**
Central Greece Deaths	6,072	6,023	6,202	6,181	6,168	5,976	6,030	6,315	6,112	5,859	5,860	6,305	6,593	5,968	6,230	6,578
Mortality	0.56	0.56	0.57	0.57	0.56	0.54	0.55	0.57	0.55	0.53	0.53	0.57	0.59	0.54	0.57	0.61
Annual Change		0	0.01	0	0	-0.02	0	0.02	-0.02	-0.02	0	0.04	0.03	-0.05	0.03	0.04
Mean Change										-0.003*						0.014**
Peloponnese Deaths	7,292	6,997	7,008	7,006	6,820	6,729	6,978	7,127	6,996	7,002	6,961	7,029	7,410	6,852	6,917	7,548
Mortality	0.67	0.65	0.64	0.64	0.62	0.61	0.63	0.65	0.63	0.63	0.63	0.63	0.67	0.62	0.63	0.7
Annual Change		-0.02	0	0	-0.02	-0.01	0.02	0.01	-0.01	0	-0.01	0.01	0.04	-0.05	0.01	0.06
Mean Change										-0.004*						0.011
East Macedonia/Thrace Deaths	6,141	6,256	6,201	6,295	6,249	6,428	6,454	6,651	6,502	6,653	6,912	6,772	7,122	7,041	7,016	7,420
Mortality	0.56	0.58	0.57	0.58	0.57	0.59	0.59	0.6	0.59	0.6	0.62	0.61	0.64	0.64	0.64	0.68
Annual Change		0.01	-0.01	0.01	-0.01	0.01	0	0.02	-0.01	0.01	0.02	-0.01	0.03	0	0	0.04
Mean Change										0.004*						0.014**
Central Macedonia Deaths	16,540	16,078	16,351	16,788	16,800	17,102	17,303	18,087	18,000	17,959	18,510	18,424	19,938	19,064	19,716	20,874
Mortality	1.52	1.48	1.5	1.54	1.54	1.56	1.57	1.64	1.63	1.62	1.66	1.66	1.8	1.73	1.8	1.92
Annual Change		-0.03	0.02	0.04	0	0.02	0.01	0.07	-0.01	-0.01	0.05	-0.01	0.14	-0.07	0.07	0.12
Mean Change										0.01*						0.05**

Year	2000	2001	2002	2003	2004	2005	2006	2007	2008	2009	2010	2011	2012	2013	2014	2015
West Macedonia Deaths	2,839	2,818	2,846	2,954	2,937	2,916	2,924	2,982	3,077	3,034	3,104	3,124	3,299	3,196	3,144	3,364
Mortality	0.26	0.26	0.26	0.27	0.27	0.27	0.27	0.27	0.28	0.27	0.28	0.28	0.3	0.29	0.29	0.31
Annual Change		0	0	0.01	0	0	0	0	0.01	0	0.01	0	0.02	-0.01	0	0.02
Mean Change										0.001*						0.007**
Thessaly Deaths	7,742	7,515	7,975	7,642	8,014	7,542	7,573	8,180	8,006	8,129	7,990	8,292	8,598	8,127	8,324	8,518
Mortality	0.71	0.69	0.73	0.7	0.73	0.69	0.69	0.74	0.72	0.73	0.72	0.75	0.78	0.74	0.76	0.78
Annual Change		-0.02	0.04	-0.03	0.03	-0.04	0	0.05	-0.02	0.01	-0.01	0.03	0.03	-0.04	0.02	0.02
Mean Change										0.003*						0.009**
Epirus Deaths	3,626	3,473	3,615	3,591	3,567	3,579	3,603	3,663	3,791	3,641	3,515	3,761	3,984	3,843	3,707	4,073
Mortality	0.33	0.32	0.33	0.33	0.33	0.33	0.33	0.33	0.34	0.33	0.32	0.34	0.36	0.35	0.34	0.38
Annual Change		-0.01	0.01	0	0	0	0	0	0.01	-0.01	-0.01	0.02	0.02	-0.01	-0.01	0.04
Mean Change										-0.0005*						0.008
North Aegean Deaths	2,628	2,472	2,472	2,571	2,460	2,413	2,440	2,450	2,323	2,395	2,349	2,263	2,507	2,303	2,357	2,488
Mortality	0.24	0.23	0.23	0.24	0.22	0.22	0.22	0.22	0.21	0.22	0.21	0.2	0.23	0.21	0.22	0.23
Annual Change		-0.01	0	0.01	-0.01	0	0	0	-0.01	0.01	0	-0.01	0.02	-0.02	0.01	0.01
Mean Change										-0.003*						0.002**
South Aegean Deaths	2,368	2,340	2,471	2,531	2,374	2,458	2,395	2,511	2,417	2,505	2,525	2,690	2,669	2,558	2,746	2,799
Mortality	0.22	0.22	0.23	0.23	0.22	0.22	0.22	0.23	0.22	0.23	0.23	0.24	0.24	0.23	0.25	0.26
Annual Change		0	0.01	0	-0.01	0.01	-0.01	0.01	-0.01	0.01	0	0.01	0	-0.01	0.02	0.01
Mean Change										0.001*						0.005**
Crete Deaths	5,514	5,346	5,481	5,609	5,324	5,529	5,633	5,746	5,560	5,434	5,573	5,692	5,879	5,580	5,792	6,162
Mortality	0.51	0.49	0.5	0.51	0.49	0.5	0.51	0.52	0.5	0.49	0.5	0.51	0.53	0.51	0.53	0.57
Annual Change		-0.01	0.01	0.01	-0.03	0.02	0.01	0.01	-0.02	-0.01	0.01	0.01	0.02	-0.02	0.02	0.04
Mean Change										-0.002*						0.013**

* Mean annual difference for the period 2000-2009

** Mean annual difference for the period 2010-2015
